# ECO/siRNA nanoparticles and breast cancer metastasis

**DOI:** 10.18632/oncoscience.200

**Published:** 2015-08-20

**Authors:** Zheng-Rong Lu, William P. Schiemann

**Affiliations:** Case Comprehensive Cancer Center, Case Western Reserve University, Cleveland, OH, USA

**Keywords:** nanoparticle, image-guided therapy, metastasis, breast cancer, EMT

Metastasis is often referred to as “The Last Frontier in Cancer Research” due to the cellular, molecular, and genetic complexities associated with this malignant behavior. Metastatic breast cancer is refractory to targeted therapeutics and chemotherapies, and as such, advanced disease states are incurable and possess a median survival of only 1-3 years. Thus, the development of safe and effective therapies against breast cancer metastasis is clearly needed to improve the overall survival of patients with this disease. This therapeutic deficit is particularly relevant to the clinical manifestations of triple-negative breast cancers (TNBCs), which lack expression of hormone receptors (estrogen receptor-α and progesterone receptor), and of the ErbB2/HER2 amplicon. Clinically, FDA-approved targeted therapies effective against TNBCs do not exist, a deficit that contributes significantly to the aggressive relapse and poor survival rates amongst women bearing TNBCs [1]. Although the molecular networks operant in mediating the metastatic progression of TNBCs remain to be fully elucidated, recent findings implicate the initiation and resolution of epithelial-mesenchymal transition (EMT) programs as prominent drivers of TNBC metastasis, and as essential promoters of their recurrence, chemoresistance, and “stemness” [2]. Thus, identifying, characterizing, and ultimately targeting fundamental effectors operant in producing EMT and metastatic phenotypes may prove to be highly effective in alleviating metastatic TNBCs.

To test the aforementioned hypothesis, we exploited the central importance of β3 integrin as a master regulator of EMT programs and metastasis in TNBCs [2]. In doing so, we loaded ECO-based nanoparticles with siRNA against β3 integrin (ECO/siβ3), whose subsequent administration to human and murine TNBCs not only abrogated their expression of β3 integrin, but also inhibited their acquisition of EMT and invasive phenotypes, as well as their growth in 3D-culture systems [3]. Moreover, we speculated that the necessity of TNBCs to upregulate their expression of β3 integrin as they transition through and ultimately complete EMT programs would provide a targetable marker to “treat” metastatic TNBCs in a preclinical therapy model. As expected, conjugating RGD peptides to ECO/siβ3 particles greatly enhanced the delivery and uptake of siRNA into TNBCs, particularly those that completed the EMT program. More importantly, treating mice with RGD-conjugated ECO/siβ3 nanoparticles dramatically inhibited the growth of primary TNBC tumors, as well as significantly reduced their metastatic burden [3]. Collectively, these findings establish targeted ECO/siRNA nanoparticles as safe, versatile, and highly effective siRNA delivery vehicles; they also implicate β3 integrin and its inactivation by ECO/siβ3 nanoparticles as an innovative potential treatment modality to alleviate post-EMT and metastatic TNBCs.

Early detection of metastatic lesions represents an essential step for effectively treating metastatic breast cancers with targeted ECO/siβ3 nanoparticles. Accordingly, we recently developed a small peptide-targeted MRI contrast agent to noninvasively image fibronectin (FN), a classical mesenchymal marker expressed by post-EMT carcinoma cells, including TNBC cells. Likewise, elevated FN expression also localizes to the tumor microenvironment and the metastatic niche [4]. After administering this novel MRI contrast agent to mice harboring TNBC tumors, we employed high-resolution MRI to detect TNBC micrometastases as small as 300 μm in diameter, which corresponds to secondary lesions that contain only a few hundred cells [5]. Thus, by combining the capabilities of early detection engendered by the targeted MRI imaging agent with the therapeutic efficacy of ECO/siβ3 nanoparticles, we have created an innovative theranostic strategy proficient in providing a personalized medicine regimen operant in eliminating metastatic TNBCs.

**Figure 1:**
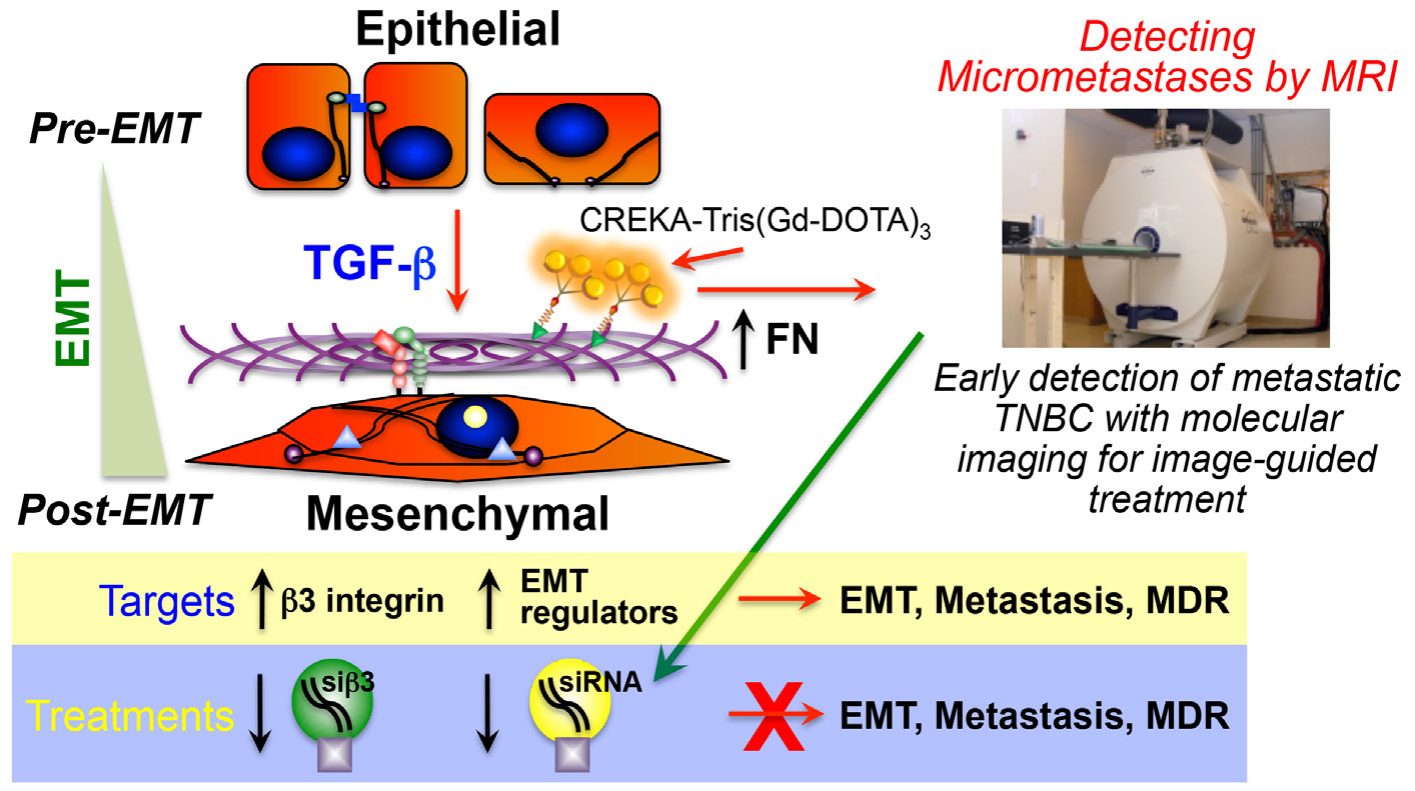
Image-guided treatment of EMT programs in metastatic breast cancers Early cancer detection by molecular imaging of the EMT marker, fibronectin (FN), and the treatment of metastatic breast cancer by inactivating β3 integrin expression with ECO/siRNA nanoparticles and, consequently, inhibiting EMT programs.

It is important to note that the dependency upon β3 integrin to drive EMT programs is not universal amongst all metastatic breast cancer cells, some of which some are reliant upon additional master regulators of EMT during metastatic dissemination [6]. Importantly, ECO is a simple and versatile platform technology that enables science and medicine to readily (i) package and deliver a variety of biological molecules, including miRNA mimics or antagonists, and (ii) target an endless array of cell types and tissue microenvironments via conjugation of cell- or tissue-specific epitopes. Accordingly, we are now expanding our efforts to target and inactivate additional drivers of EMT programs using ECO nanoparticles loaded with specific siRNA or miRNA. Moreover, targeting the EMT program provides a unique opportunity to prevent the expansion and self-renewal of breast cancer stem cells (CSCs), thereby exhausting the CSC pool and promoting durable complete pathological responses(pCRs) in patients with metastatic disease. Successful inactivation of the EMT program will also thwart the appearance of disease recurrence and the development of chemoresistance. Indeed, EMT gene signatures are surprisingly reminiscent of those recently associated with metastatic dormancy, suggesting an intriguing association between EMT programs and dormant phenotypes. As such, the administration of ECO/siβ3 nanoparticles or their derivatives may provide a novel approach to eliminate dormant micrometastases, which currently escape clinical detection and underlie disease recurrence. Similarly, post-EMT cells are generally non-proliferative and, consequently, are insensitive to chemotherapies that target cell proliferation or DNA damage pathways. Thus, while reverting the EMT phenotype may restore the proliferative capacity of disseminated, this counterintuitive sequence of events could then be harassed as a means to sensitize TNBCs to traditional standard-of-care agents. Future studies need to address these possibilities, as well as expand the utility of ECO-based theranostics to (i) other genetically distinct TNBC and breast cancer subtypes; (ii) additional human malignancies, particularly those with a propensity to acquire EMT phenotypes (e.g., lung, ovarian, pancreas, etc.); and (iii) other fibrotic or hyperproliferative disorders.
